# Addressing the quality challenge of a human biospecimen biobank through the creation of a quality management system

**DOI:** 10.1371/journal.pone.0278780

**Published:** 2022-12-30

**Authors:** Marie-Dominique Servais, Florence Galtier, Agathe Nouvel, Sandra Rebuffat, Jonas Laget, Anne Géan, Nicolas Provost, Frédéric Lorcy, Valérie Rigau, Guilhem Couderc, Philippe Géraud, David Nocca, Nicolas Builles, Nathalie De Préville, Anne-Dominique Lajoix

**Affiliations:** 1 Servier, Suresnes, France; 2 INSERM, Clinical Investigation Center 1411, St Eloi Hospital, University Hospital of Montpellier, Montpellier, France; 3 Department of Endocrinology, Lapeyronie Hospital, University Hospital of Montpellier, Montpellier, France; 4 Biocommunication in Cardio-Metabolism (BC2M), University of Montpellier, Montpellier, France; 5 Biological Resources Center, Anatomy and Cytology Laboratory, University Hospital of Montpellier, Montpellier, France; 6 Biological Resources Center, Tissue Bank, University Hospital of Montpellier, Montpellier, France; 7 Department of Digestive Surgery, University Hospital of Montpellier, Montpellier, France; College of Food Technology, Dr. Punjabrao Deshmukh Krishi Vidyapeeth, Akola, INDIA

## Abstract

**Background:**

The objective of the COMET (COllection of MEtabolic Tissues) biobank project is to create a high-quality collection of insulin-sensitive tissues (liver, muscle, adipose tissues, and epiploic artery) and blood sample derivatives (plasma, serum, DNA and RNA), collected from 270 grade 2–3 obese patients undergoing bariatric surgery. Relevant data on patient such as clinical/biological characteristics and sample handling are also collected. For this, our aim was to establish a Quality Management System (QMS) to meet the reliability and quality requirements necessary for its scientific exploitation.

**Materials and methods:**

The COMET QMS includes: (1) Quality Assurance to standardize all stages of the biobanking process, (2) Quality Controls on samples from the first patients included in order to validate the sample management process and ensure reproducible quality; and 3) “in process” Quality Controls to ensure the reliability of the storage procedures and the stability of the samples over time.

**Results:**

For serum and plasma, several corrective actions, such as temperature handling and centrifugation conditions, were made to the protocol and led to improvement of the volume and quality of samples. Regarding DNA, all samples evaluated achieved a satisfactory level of purity and integrity and most of them yielded the required DNA quantity. All frozen tissue samples had RNAs of good purity. RNA quality was confirmed by RIN, achieving values in most cases over 7 and efficient amplification of housekeeping genes by RT-qPCR, with no significant differences among samples from the same tissue type. In the “in process” Quality Controls, DNA, RNA, and histological integrity of tissues showed no differences among samples after different preservation times.

**Conclusion:**

Quality Control results have made it possible to validate the entire biobank process and confirm the utility of implementing QMS to guarantee the quality of a biospecimen collection.

## Introduction

The development of human tissue biobanks is now considered as an indispensable tool for the progression of biomedical research and the development of new therapeutic strategies in personalized medicine (population stratification, biomarker discovery,…) [1]. At present, one of the challenges of human biospecimen biobanks is to provide quality-assured materials as well as accurate and reliable associated data (biospecimen are defined as tissues or blood derivatives removed from the patient). There is a recognized need to develop more standardized and harmonized technical procedures for the constitution of biobanks, especially in terms of biospecimen collection and associated data. In this context, the purpose of the COMET (COllection of MEtabolic Tissues) biobank project [2] is to create a high-quality collection of insulin-sensitive tissues (liver, muscle, adipose tissues and epiploic artery) and blood derivatives (DNA, RNA, plasma and serum) collected from grade 2–3 obese patients (body mass index [BMI] ≥ 35 kg/m^2^) undergoing bariatric surgery. The overarching goal of this biobank is to promote translational and pharmaceutical research in the field of type 2 diabetes (T2D) and related metabolic disorders.

Multiple preanalytical factors can affect the integrity of human biospecimens and may thus impact downstream molecular applications. Surgical resection methods, duration and conditions of transport, time and temperature prior to snap-freezing can lead to metabolic, biochemical, and physical stresses known as warm ischemia [3]MA. These factors can also impact the long-term integrity of samples after several years of storage in the biobank (samples are defined as aliquots of tissues or blood derivatives stored in the biobank). Therefore, the quality of human samples and the traceability of sample-related information must be efficiently standardized by the creation of specific procedures to be applied during the biobanking [4].

In order to achieve this, the COMET biobank constitution process was standardized using a Quality Management System (QMS), with two components: Quality Assurance and Quality Controls. The QMS included quality controls of samples at microscopic, macroscopic and molecular levels and validation of the sample management process on the first patients included in the clinical trial. Quality controls were carried out regularly to verify that the quality of samples was maintained over time. In addition, special attention was paid to the integrity and traceability of associated data.

## Materials and methods

### Ethical consideration, study population and associated data

The COMET biobank project, initiated in 2015, was performed in compliance with French and international regulations, such as the provisions relating to biomedical research, the Public Health Act no. 2004–806, the Data Protection Act and the Bioethics Act. It also followed the Declaration of Helsinki and Good Clinical Practice, and was approved by the French National Agency for the Safety of Medicines and Health Products (ANSM) and an Ethics Committee. The clinical trial was registered on August, 10^th^ 2016 under the number NCT02861781 on www.clinicaltrials.gov (https://clinicaltrials.gov/ct2/show/NCT02861781).

Candidate patients for the biobank were asked to sign a written informed consent form covering biospecimen and associated data storage of up to 25 years and their use in research programs, including genomics. They received a copy of both the information document and signed consent form and were informed of their right to revoke their consent at any time, without incurring any consequences for their medical follow-up. Patients were definitively included in the clinical trial if negative results for the serological tests were obtained (HIV, hepatitis B and C). In case of positive serology, the patient was replaced.

The patients included in the COMET biobank were between 18 and 65 years of age and had grade 2–3 obesity (BMI ≥ 35 kg/m^2^) requiring sleeve gastrectomy. They were stratified by age and metabolic status in three groups: insulin sensitivity (Homeostatic Model Assessment of Insulin Resistance [HOMA-IR] < 3), insulin resistance without T2D (HOMA-IR ≥ 3), or T2D (according to American Diabetes Association (ADA) criteria [5]). For each patient, biopsies from insulin-sensitive tissues (subcutaneous and visceral adipose tissues, liver, muscle and epiploic artery tissues) were collected during the gastrectomy. Blood derivative biospecimens (DNA, plasma and serum) were also collected from whole blood sampled during the study. By the end of the project, the intention is for the biobank to have collected approximately 70,000 samples (from a total of 270 patients).

In this project, patients were largely characterized by both clinical data (medical history, ongoing treatments, quality-of-life questionnaires, family history of obesity and diabetes, eating behaviors, physical activity, lifestyle, weight, waist circumference, vital signs) and biological data (fasting glucose, insulin, C-peptide, free fatty acids, liver function tests, lipid profile, HbA1c, creatinine). The data collected were pseudonymized and entered in an electronic Case Report Form (eCRF), enabling its real-time quality control. The eCRF (duly declared to the French Data Protection Authority) was prepared using the Capture System (Clinsight^®^), which complies with Food and Drug Administration (FDA, USA) recommendations for the management of clinical trials and electronic signature and standards.

### Training of team members

Team members involved in the biocollection were qualified, empowered and trained at the initiation of the project, and then re-accredited annually. Specific training was given on liquid nitrogen safety conditions and on Tumorotek^®^ and Clinsight^®^ software. Concerning biosafety and biohazard management, several actions have been implemented to guaranty the safety of team members in the surgical block and during handling of biospecimen of human origin: 1) training for exposure to blood and biological products; 2) use of individual protective equipment, such as masks, gloves, charlottes, in addition to wearing surgical attire at the surgical block; 3) recruitment of patients with negative serologies (HIV, HBV and HCV); 4) follow-up of each team member by occupational health-care professionals, and check for valid vaccination certificate. In case of accidental contact with blood samples, serological controls will be performed few days after the contact and then 6 months after.

The biobank is located at the Biological Resource Center (BRC) of the University Hospital of Montpellier, France (BB-0033-00031) [6].

### Quality management system

The BRC, where the biobank is hosted, follows international ISO 9001 rules for quality management and more specifically ISO20387 dedicated on Biobanking. The COMET biobank also follows the ICH Q10 guideline for pharmaceutical quality system and the Q9 guideline for quality risk management.

At the beginning of the project, a QMS was adopted in order to guarantee the reproducibility, traceability and high-quality preservation of samples and associated data. For Quality Assurance, tools such as a technical protocol and operating procedures were established to standardize all stages of the sample collection process. For Quality Control, validation and verification of the sample management process ensured that they were of the desired quality. For each patient, a traceability document named “Sample tracking sheet” in paper format was created and used to record all information related to sample collection, processing, transfer and storage (Table 1). Information were recorded in the software application Tumorotek^®^ (Ministry of Health, France), used to manage biospecimens storage and handling data. Sample tracking sheets were stored at the BRC and could be used as a backup in case of computer problems.

**Table 1:** Relevant biospecimen information recorded on the sample tracking sheet.

	For all biospecimens
**All biospecimens**	• **Number of samples obtained** • **Conformity of the biospecimens (Yes/No, if No: reason)** • **Date and time of storage (-80°C) at the biobank center** • **Sample/storage box ID number** • **Number of biospecimens stored at -80°C** • **Name of the operator**
	**For specific biospecimens**
**Serum and plasma**	• Date of blood draw • Blood sample departure and arrival times • Dry shipper ID number • Dry shipper temperature on departure and arrival • Lag time between venepuncture and centrifugation • Lag time between centrifugation and freezing • Presence of hemolysis, clotting
**DNA**	• Date/time of blood draw • Date of sample transfer (blood sample, DNA sample) • Date and time of storage (blood sample, DNA sample) • Date of DNA extraction from blood • Transfer at 4°C of extracted DNA to the freezer • Lag time between venepuncture and DNA extraction • A260/280 ratio, DNA concentration, DNA quantity, volume/tube • Time between blood sampling and DNA extraction (< 28 days)
**Frozen tissues**	• Tissue localization of biopsy • Time of biopsy • Handling duration prior to freezing • Time at departure from surgery room/arrival at biobank center • Dry shipper temperature on departure and arrival

The consistency between the data contained in the Sample tracking sheets and in the Tumorotek® database was regularly verified. Data monitoring were performed to test for inconsistencies in patient clinical data. Our banking system was tested during storage with monitoring audits performed by partners consisting of random file inspections. When sample transfers occurred, queries between partner and our own database were tested, along with physical control. The rate of errors detected through this system was less than 2%.

To avoid sample-handling errors during collection, cryotubes and storage boxes were pre-labelled with a unique serial number generated with a unique alphanumeric code to allow precise management of sample IDs. The cryotube caps were color-coded to easily distinguish each tissue type and sample.

### Validation and verification of the sample management process

Critical parameters (such as date/time of sampling, preparation duration, number and weight of samples…) were defined for each type of tissue (Table 2) and described in a specific protocol. The ranges defined for these critical parameters (specifications) had to be followed to ensure optimal sample quality.

**Table 2:** Critical parameters and acceptance criteria for sample management process validation and verification.

Tissues (number of patients assessed)	Critical parameters	Objectives	Consequences if not achieved	Acceptance criteria
**Plasma (first 16 patients)**	**Centrifugation conditions**	**Compliance with protocol/operating procedure** 15 min/2000g/4°C (optimized at 15 min/2000g/10°C)	Poor separation (clotting)	**Volume obtained for expected number of samples**
**Serum (first 16 patients)**	**Centrifugation conditions**	**Compliance with protocol/operating procedure** 10 min/1000g/4°C (optimized at 15 min/2000g/10°C)	Poor separation (clotting) Post-coagulation	**Number of samples**
**Genomic DNA (first 27 patients)**	**Extraction Conditions**	**Compliance with protocol/operating procedure** Within 28 days of sampling	Insufficient quantity of DNA Poor quality DNA	**Number of samples obtained** **Quantity and purity:** 100–500 μg DNA/sample A260/280 ratio ≥1.8–2
Frozen biopsies* (first 5 patients)	**Handling conformity** **Handling time**	**Compliance with protocol//operating procedure** **Determination of maximum handling time, four conditions tested:** **-** All tissues: ≤10, ≤15 and ≤20 minutes before freezing - Adipose tissues only: ≤25 minutes before freezing	Insufficient quantity of RNA Poor quality RNA Impact on downstream gene expression analyses	**RNA purity and integrity** A260/280 ratio ≥2 RIN ≥7 28S/18S ratio: 1.9–2.1 **Determination of maximum handling time** **RT-qPCR analyses:** RT-qPCR analyses: measurement of gene expression levels (Ct values for each housekeeping gene)

* Biopsies: subcutaneous adipose tissue (umbilical region), visceral adipose tissue (greater omentum, gastrosplenic region), liver (segment II or III, left lobe), abdominal skeletal muscle (rectus abdominis).

Samples of consecutive operated patients were used to validate the sample management process (sample collection, preparation, transportation and storage) for the whole project. The validated specifications were then included in the technical protocol and operating procedures for application to following patients. All non-conformities regarding the technical protocol had to be recorded on the Sample tracking sheet and in Tumorotek®.

Quality of samples was (and will be) verified on randomly selected samples every two years to ensure the continued validity of the sample management process. If the quality did not meet the predefined criteria, corrective actions were eventually required before continuing with the biobanking, in order to ensure a robust and reproducible process.

### DNA collection

Following patient consent, blood samples were collected in PAXgene Blood DNA tubes (8.5 mL, Qiagen, Germany), inverted 8–10 times immediately after sampling, transported to the BRC at room temperature and stored at +4°C. DNA was isolated using the PAXgene Blood DNA kit (Qiagen, Germany) according to the manufacturer’s instructions, no later than 28 days after sampling. The concentration and purity of DNA solutions were determined using the NanoDrop One/One spectrophotometer (Thermo Fisher Scientific, USA), before storage at -80°C. We measured the A260/280 ratio as it reflects the presence of the most abundant contaminants, such as proteins, phenol or other compounds that absorb strongly at or near 280 nm. The A260/280 ratio to be reached for DNA was between 1.8–2 (Table 2). Extracted DNA was then distributed in cryotubes for storage at -80°C. After cession, the partner can thaw DNA samples to prepare several aliquots of the desired quantity, in order to limit freeze- thaw cycles and preserve their integrity.

DNA (1 μg per well) was separated on 0.8% agarose gel in the presence of Gel Red (Biotium, Fremont, CA, USA) and visualized on a E-Box Vilbert Lourmat imager. DNA degradation was determined visually by the disappearance of the high molecular weight band located at the top of the gel, corresponding to intact DNA.

### Serum and plasma collection

Blood samples for plasma (16 mL, EDTA K2E tubes, Becton Dickinson, UK) and serum (25 mL after optimization, dry tubes, Becton Dickinson, UK) were drawn under fasting conditions, on the day of surgery and during follow-up consultations (3 and 12 months later). Following the results of the sample management process (see Results), the final technical procedure for plasma and serum extraction was established. Following venipuncture, blood samples were transported to the BRC at room temperature prior to processing instead of 4°C. Tubes were then centrifuged for 15 minutes at 2000 g and 10°C, within a maximum of 2 hours after collection for plasma and within 30 minutes to 2 hours for serum. Plasma and serum were then aliquoted into 500 μL volumes and directly frozen at -80°C.

### Tissue samples collection

Biopsies were performed during the bariatric surgery. Their location, operating procedure and quantity of tissue taken were established by the surgeon and reproduced as far as possible at each surgery.

Biopsies were cut into small pieces (at least 50 mg for adipose tissues, 20 mg for liver and muscle and 3–4 cm for epiploic artery tissues) using a sterile single-use scalpel in a Petri dish placed on ice. Following sample management process (see Results), the maximum time between biopsy resection and freezing was defined as 15 minutes for liver and muscle and 20 minutes for adipose tissues. Each tissue aliquot was deposited, with no additives, into a pre-labelled cryotube. The cryotubes were then immediately snap-frozen by immersion in a cryogenic tank and transferred in a liquid nitrogen dry shipper, the inner wall of which is covered by a porous material that absorbs a certain volume of liquid nitrogen. The container was saturated with cold nitrogen gas to maintain a stable temperature at -140°C, constantly registered by a sensor located in the top of the container.

### Long-term storage of samples

The storage facility selected was the Tissue Bank of the University Hospital of Montpellier–part of the BRC. It received ANSM authorization for the medical use of tissues and cells and is ISO-9001 and NF-S-96900 certified. Access to the storage area was controlled and secured with a digital code. Samples were stored in latest generation two-engine electrical -80°C freezers. The BRC freezers were all qualified according to a GMP-like protocol. They were secured, continuously monitored using a wireless temperature data logger (Oceasoft, France) and equipped with a remote alarm in case of temperature deviation (above -70°C). On-calls were planned for nights and weekends in case of freezer malfunction, for repair or transfer to the emergency freezers. The storage room was equipped with a CO_2_ ramp for gas injection into the freezers in the event of malfunction, allowing time for intervention.

### RNA extraction and quality control

20 mg of frozen skeletal muscle and liver and 50 mg of frozen adipose tissues were homogenized in QIAzol Lysis Reagent according to the manufacturer’s recommendations (Qiagen, Germany) using gentleMACS Dissociator^TM^ (Miltenyi Biotec, Germany). RNA extraction was then performed following the recommendations of the RNeasy (Lipid Tissue) Mini Kit (Qiagen, Germany) with DNase digestion in a QIAcube device. RNA concentration and purity were evaluated using DropSense^TM^ 96 (Trinean, USA). Nucleic acid integrity was evaluated using the Agilent RNA 6000 Nano Kit on a Bioanalyzer 2100 electrophoresis system (Agilent Technologies Inc., USA). The RNA Integrity Number (RIN), 28S/18S ribosomal RNA ratio, and DV_200_ [7] were determined using the software provided by the manufacturer. No RNA quality assessments were performed on epiploic artery biospecimens.

### Reverse transcription and quantitative PCR (RT-qPCR)

For reverse transcription (RT), 0.5 μg of total RNA (1 μg for liver RNA) was reverse-transcribed using the High-Capacity cDNA Reverse Transcription Kit with RNase Inhibitor (Thermo Fisher Scientific, USA) according to the manufacturer’s instructions. Then, 10 ng (20 ng for liver) of cDNA was used for qPCR performed with a 7500 Fast Real-time PCR System (Thermo Fisher Scientific, USA) using the QuantiFast SYBR^TM^ Green PCR Kit (Qiagen, Germany) according to the manufacturer’s instructions. The PCR cycling was performed with KiCqStart^®^ SYBR^®^ Green primers (Sigma, USA) (See S1 Table) for a panel of six housekeeping genes widely used according to the following conditions: 95°C for 5 minutes (initial denaturation), then 40 cycles at 95°C for 10 seconds followed by 60°C for 30 seconds and 72°C for 15 seconds. Following amplification, PCR products underwent analysis of melting curve, linearity, and slope of standard curve using 7500 Software (Thermo Fisher Scientific, USA) to confirm the amplification specificity. All RT-qPCR assays were performed in duplicate.

### Histochemistry

Frozen tissues were embedded in OCT, cut on cryostat (5 μm section) and stained with hematoxyline-eosine, according to standard protocols. Alternatively, tissue samples were thawed, fixed in 10% formalin for at least 24h. After dehydration and embedding in paraffin, sections of 3 μm were cut using a microtome and stained with hematoxyline-eosine. Tissue integrity was analyzed on a LEICA DM 2500 microscope. Representative images were taken with magnification of 200.

### Statistical analysis

For some experimental values, the mean of n experiments ± standard deviation (SD) was calculated. Statistical analysis was performed with GraphPad Prism 8.0.2 software using Student’s t test or one-way ANOVA when applicable.

## Results

### Serum and plasma quality

For serum and plasma, centrifugation conditions of blood were chosen as critical parameter for quality of samples (Table 2). Other parameters could also impact their quality, such as volume of blood, tube transport temperature, conditions of aliquoting (Table 3).

**Table 3:** Initial and optimized conditions for plasma and serum biospecimen preparation and the corresponding findings.

Step	Initial conditions (patients C01 to C03)	Conditions after optimization (patients C04 to C16)
Blood sample volume	Plasma: 16 mL EDTA tubes Serum: 20 mL dry tubes	Plasma: 16 mL EDTA tubes Serum: 20 mL dry tubes
Transport	In a cooler (4°C)	**Immediately, at room temperature**
Time to centrifugation	Plasma: immediately Serum: 30 minutes after sampling	Plasma: **within 2 hours** Serum: **between 30 minutes and 2 hours after sampling**
Centrifugation conditions	15 minutes/2000g/4°C (Plasma) 10 minutes/1000g/4°C (Serum)	**Unique centrifugation protocol for both plasma and serum**: **15 minutes/2000g/10°C**
Aliquoting	Plasma: 15 samples, Serum: 20 samples On ice, in 500 μl volumes, then frozen at -80°C	Plasma: 15 samples, Serum: 20 samples On **refrigerating rack,** in 500 μl volumes, then frozen at -80°C
**Results**	Number of patients with a compliant number of samples: Plasma: 0/3 patients Serum: 0/3 patients, **clotting in supernatant** after centrifugation	Number of patients with a compliant number of samples: Plasma: 8/13 patients Serum: 4/13 patients, **no clotting** in supernatant after centrifugation **➢ blood sample volume increased from 20 mL to 25 mL for the remaining collections**

For the first three patients included in the biobank, cases of hemolysis and clotting were observed during biospecimen processing. The expected volume (i.e. the expected number of tubes of 500 μl) of plasma and serum was not obtained, and serum extraction was hindered by an impaired clotting process in the tube resulting from storage at 4°C (Table 3). Consequently, the technical protocol was optimized with changes in temperature handling and centrifugation conditions. Following these corrective actions, serum and plasma biospecimens from the following thirteen patients showed less hemolysis and clotting and were thus more compliant to acceptance criteria (Table 3). The initial volume of blood to be taken for serum extraction (20 mL) was increased to 25 mL to achieve the expected number of tubes, thereby leading to a clinical protocol amendment. Due to modifications of processing conditions, blood derivatives issued from the first three patients won’t be used for future studies.

### DNA quality

The critical parameter identified was the extraction conditions, which could impact quantity and quality (purity and potential fragmentation) of DNA (Table 2). DNA from the first 27 patients were extracted and controlled in order to validate the sample management process.

### DNA purity and integrity

**Fig 1:**
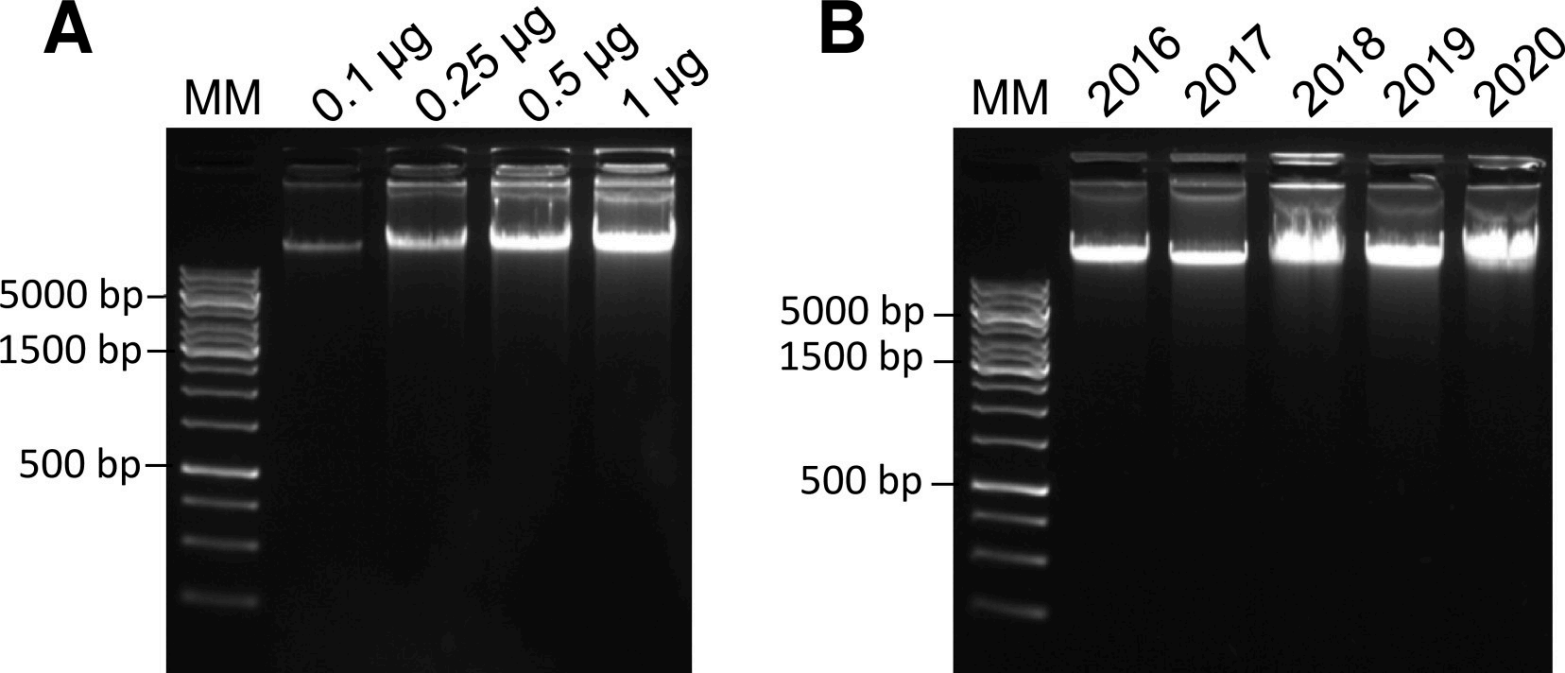
DNA integrity according time of storage in the biobank. After extraction, DNA was separated on 0.8% agarose gel and visualized by Gel Red staining on an imager. (A) Electrophoresis of increasing amounts of DNA after extraction from PAXgene tube. (B) Electrophoresis of DNA after extraction and storage at –80°C. The year in which the extraction is carried out is indicated. MM: molecular markers.

The mean DNA yield obtained from one PAXgene tube of 8.5 mL was 496.7 ± 366.6 μg, which allows DNA preparations above 100 μg/vial for 22 out of the 27 samples (81.7%) while four were comprised between 60.0 and 98.3 μg/vial, and one preparation did not provide DNA (Table 4; n = 27). However, as a very small amount of genomic DNA is sufficient to conduct common molecular analyses [8–10], a minimum quantity of 40 μg DNA per vial was judged acceptable. If DNA levels are too low, a clinical protocol amendment allows an additional sampling at the M3 or M12 visit. IN addition, DNA yields varied between patients, but we found no correlation between the level of DNA and white blood cell count (which are the source of blood DNA) measured one month later at the time of surgery. All DNA samples achieved a satisfactory level of purity, with a mean A260/280 ratio = 1.85 ± 0.02. Migration of increasing amounts of DNA on agarose gel evidenced good quality with no traces of fragmentation (that induces the appearance of a smear instead of the high molecular weight band at the top of the gel) after the extraction process (Fig 1A).

**Table 4:** Quality metrics of DNA isolated from blood. The concentration and purity of the DNA samples isolated from the patients’ blood were evaluated based on spectrophotometric measurements at 260 nm and 280 nm.

Patient no.		Time to extraction (days)		Concentration (μg/mL)		DNA per cryotube (μg)		Volume per cryotube (μL)		A260/280 purity ratio		Number of DNA samples obtained		Total DNA(μg/patient)	
C0001		20		847		**100**		118		**1.87**		**8**		800	
C0002		19		1260		**126**		100		**1.88**		**10**		1260	
C0003		15		1304		**130.4**		100		**1.86**		**10**		1304	
C0004		13		450		**112.5**		250		**1.86**		**4**		450	
C0007		17		959		**106.6**		111		**1.91**		**9**		959.4	
C0008		17		114		**114.7**		1000		**1.84**		**1**		114.7	
C0009		22		417		**104.3**		250		**1.86**		**4**		417.2	
C0010		22		778		**111.2**		142		**1.87**		**7**		778.4	
C0011		10		253		**126.8**		500		**1.85**		**2**		253.6	
C0012		20		465		**116.4**		250		**1.86**		**4**		465.6	
C0013		13		289		**144.8**		500		**1.86**		**2**		289.6	
C0014		8		182		**182.1**		1000		**1.84**		**1**		182.1	
C0015		8		483		**120.9**		250		**1.86**		**4**		483.6	
C0016		6		132		**116.3**		500		**1.85**		**2**		232.6	
C0017		6		258		**129.4**		500		**1.85**		**2**		258.8	
C0018		15		778		**111.2**		142		**1.88**		**7**		778.4	
C0032		13		171		**85.9**		500		**1.85**		**2**		171.8	
C0033		8		211		**105.6**		500		**1.83**		**2**		211.2	
C0035		6		215		**107.9**		500		**1.83**		**2**		215.8	
C0036		6		1024		**102.4**		100		**1.86**		**10**		1024	
C0037		6		334		**111.6**		333		**1.86**		**3**		334.8	
C0038		6		196		**98.3**		500		**1.83**		**2**		196.6	
C0045		13		476		**119**		250		**1.86**		**4**		476	
C0047		13		-		**-**		-		**-**		**-**		-	
C0048		13		130		**60**		500		**1.83**		**2**		120	
C0050		8		1042		**104.2**		100		**1.86**		**10**		1042	
C0051		8		146.0		**73.2**		500		**1.80**		**2**		146.4	
Mean		12.3		496.7		**112.4**		365.2		**1.85**		4.5		**498.7**	
SD		5.3		366.6		**22.1**		245.1		**0.02**		3.1		**362**	

### DNA quality over time

The quality of DNA was also followed over time, 6 to 45 months after extraction and storage at -80°C. Mean concentration of DNA after destocking reached 330.1 ± 124.9 μg/mL as compared to 308 ± 121.8 μg/mL measured at the time of extraction (P = 0.3, non-significant (NS)) (Table 5). In addition, no traces of DNA fragmentation could be observed regardless the preparation dates of the samples and the time of storage in the biobank (Fig 1B).

**Table 5:** Quality metrics of DNA isolated from blood after destocking from the biobank. The concentration and purity of the DNA samples isolated from the patients’ blood were evaluated based on spectrophotometric measurements at 260 nm and 280 nm.

Patient no.	Time to extraction (days)	Extraction year	A260/280 purity ratio after extraction	Concentration after extraction (μg/mL)	Delay before Destocking (months)	Concentration after destocking (μg/mL)
C0147	7	2016	1.85	**223.8**	45	**153.2**
C0248	19	2017	1.85	**87.5**	40	**108.2**
C0792	28	2017	1.89	**562.7**	36	**521.8**
C0875	12	2017	1.86	**289.8**	33	**292.7**
C1025	20	2018	1.88	**304.7**	29	**395.8**
C1219	11	2018	1.85	**280.2**	23	**289.1**
C1283	20	2018	1.87	**413.4**	20	**411.3**
C1284	20	2018	1.87	**388.4**	20	**435.2**
C1437	21	2019	1.86	**239.9**	17	**429.9**
C1498	22	2019	1.85	**260.1**	16	**266.1**
C1506	7	2019	1.85	**232**	14	**241.7**
C1651	14	2020	1.86	**413.9**	6	**416**
Mean	17	/	1.86	**308**	/	**330.1**
SD	6.5	/	0.01	**121.8**	/	**124.9**

### Frozen tissue quality

For frozen tissues, two critical parameters were identified: handling conformity and handling time of samples, both factors being able to impact RNA quantity and quality (Table 2). RNA was extracted from the first five patients included in the biobank (after a short period of storage), in order to validate the sample management process.

### RNA purity and integrity

**Fig 2:**
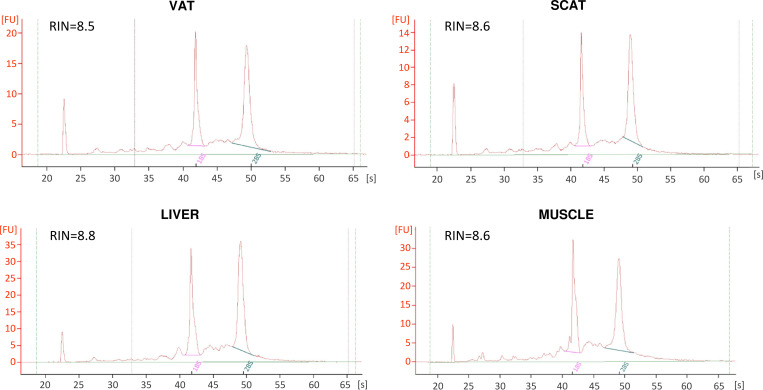
Electropherograms of RNA samples obtained for the four tissue types. Electropherograms were obtained on RNA samples using 2100 Bioanalyser and RNA 6000 Nano chips. Typical electrophoretic traces and RIN were shown for VAT, SCAT, liver, and muscle extracted in 2016. SCAT, sub–cutaneous adipose tissue; VAT, visceral adipose tissue.

Following extraction, we showed that all RNA samples had an A260/280 ratio between 1.88 and 2.09, with a median of 1.98 for subcutaneous adipose tissue (SCAT), 2 for visceral adipose tissue (VAT), 2.07 for liver and 2.05 for muscle (Table 6; n = 57). Overall, 36 out of the 57 samples had the theoretical expected A260/280 ratio of 2.0 or greater, indicating that 63.2% of RNA samples were relatively free of proteins. In addition, 17/18 SCAT RNA samples, 16/16 VAT samples, 11/12 muscle samples, and 6/11 liver samples displayed a satisfactory RIN ≥ 7 (50/57 samples) (Tables 2 and 6). As previously described [11], RIN from liver were significantly lower than other tissues (P<0.01 versus SCAT; P<0.001 versus VAT; P<0.01 versus muscle), with four samples having a RIN between 6.7 and 6.9, and only one below 6.7. Representative electrophoregrams obtained for the four different tissues were shown in Fig 2. In contrast, only one RNA sample out of the 57 tested reached the target 28S/18S rRNA ratio of 1.9–2.1 (Table 6), as we previously observed [11].

**Table 6:** Quality metrics of RNA isolated from frozen tissues obtained from the first five patients included. Absorbance at 260 and 280 nm, RIN and 28S/18S ratio were assessed for each RNA sample isolated from frozen SCAT, VAT, muscle, and liver. Mean ± SD, median, and ranges for each type of RNA are shown. The number of samples (and percentage) in the optimal value of purity (A260/280 ≥ 2) and integrity (RIN ≥ 7, 28S/18S ratio: 1.9–2.1) is also presented.

		SCAT	VAT	MUSCLE	LIVER	All
	n	18	16	12	11	57
**A260/280**	Mean ± SD	1.98 ± 0.03	2 ± 0.04	2.03 ± 0.04	2.07 ± 0.02	2.01 ± 0.05
	Median	1.98	2.00	2.05	2.07	2.01
	Min—Max	1.93–2.04	1.88–2.04	1.95–2.07	2.01–2.09	1.88–2.09
*A260/280 ≥ 2*	*n (%)*	*6 (33*.*3)*	*10 (62*.*5)*	*9 (75)*	*11 (100)*	*36 (63*.*2)*
*A260/280 ≥ 1*.*8*	*n (%)*	*18 (100)*	*16 (100)*	*12 (100)*	*11 (100)*	*57 (100)*
**RIN**	Mean ± SD	7.75 ± 0.49	7.83 ± 0.51	7.76 ± 0.54	6.82 ± 1.13	7.59 ± 0.76
	Median	7.65	7.85	7.5	7.2	7.6
	Min—Max	7.5–8.7	7.0–8.8	6.9–8.6	3.6–8.1	3.6–8.8
*RIN ≥ 7*	*n (%)*	*17 (94*.*4)*	*16 (100)*	*11 (91*.*7)*	*6 (54*.*5)*	*50 (87*.*7)*
*RIN [6; 7]*	*n (%)*	*1 (5*.*6)*	*-*	*1 (8*.*3)*	*4 (36*.*4)*	*6 (10*.*5)*
*RIN < 6*	*n (%)*	*-*	*-*	*-*	*1 (9*.*1)*	*1 (1*.*8)*
**28S/18S**	Mean ± SD	1.41 ± 0.21	1.27 ± 0.17	1.05 ± 0.11	1.01 ± 0.41	1.22 ± 0.28
	Median	1.4	1.2	1	1	1.2
	Min—Max	1.0–1.9	1.0–1.6	0.9–1.2	0–1.7	0–1.9
*28S/18S *: *1*.*9–2*.*1*	*n (%)*	*1 (5*.*6)*	*-*	*-*	*-*	*1 (1*.*8)*

### Handling time for tissue preparation

**Fig 3:**
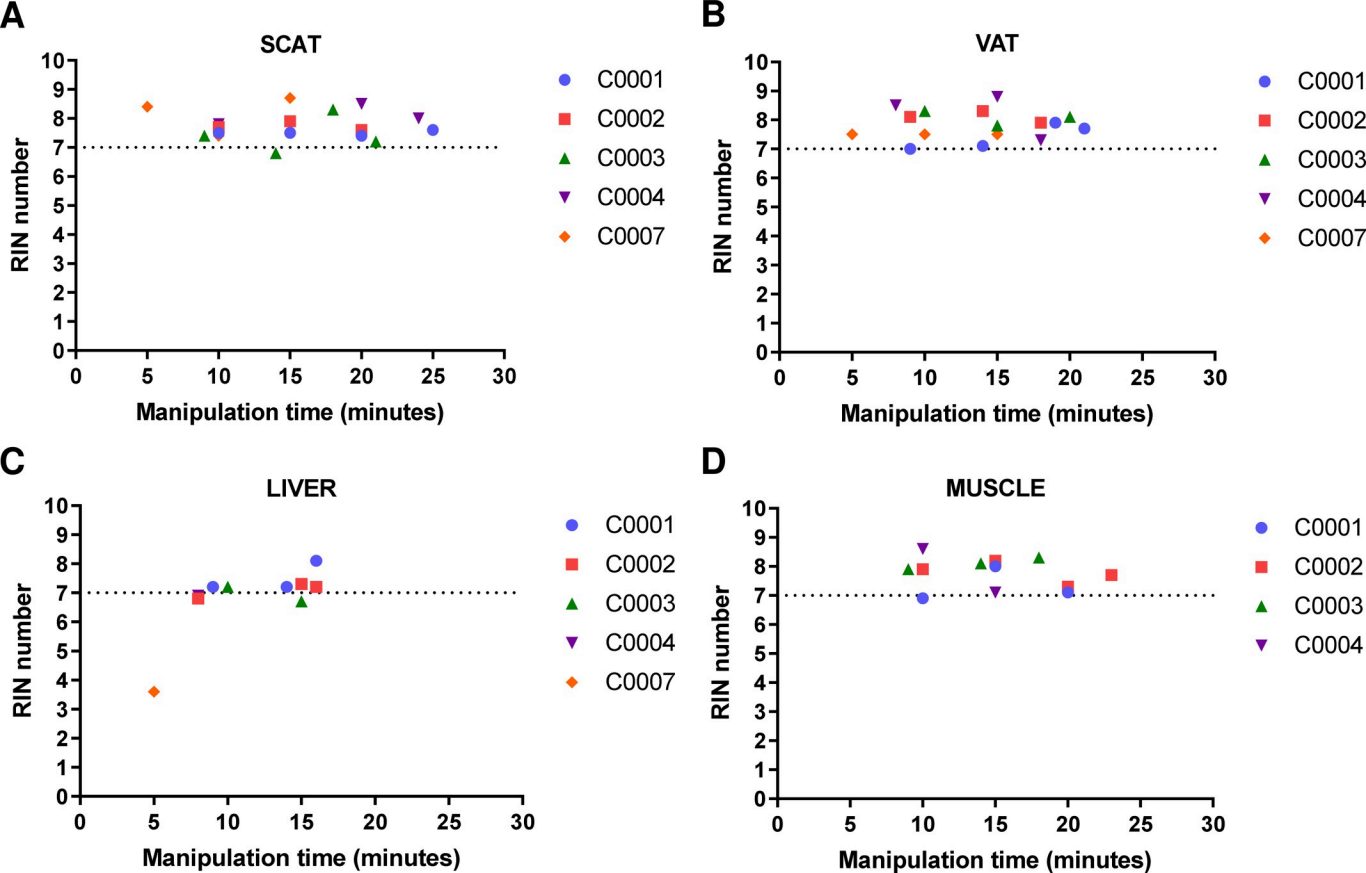
RIN according to handling time for tissue preparation for the first five patients. RNA was extracted from frozen tissues. RIN was shown according to handling times of liver, muscle, SCAT and VAT samples resected from the first five patients, before snap–freezing. The handling times between tissue resection and snap–freezing were: ≤ 10, ≤ 15 and ≤ 20 minutes (all tissues) and ≤ 25 minutes (adipose tissues only). SCAT, sub–cutaneous adipose tissue; VAT, visceral adipose tissue.

**Fig 4:**
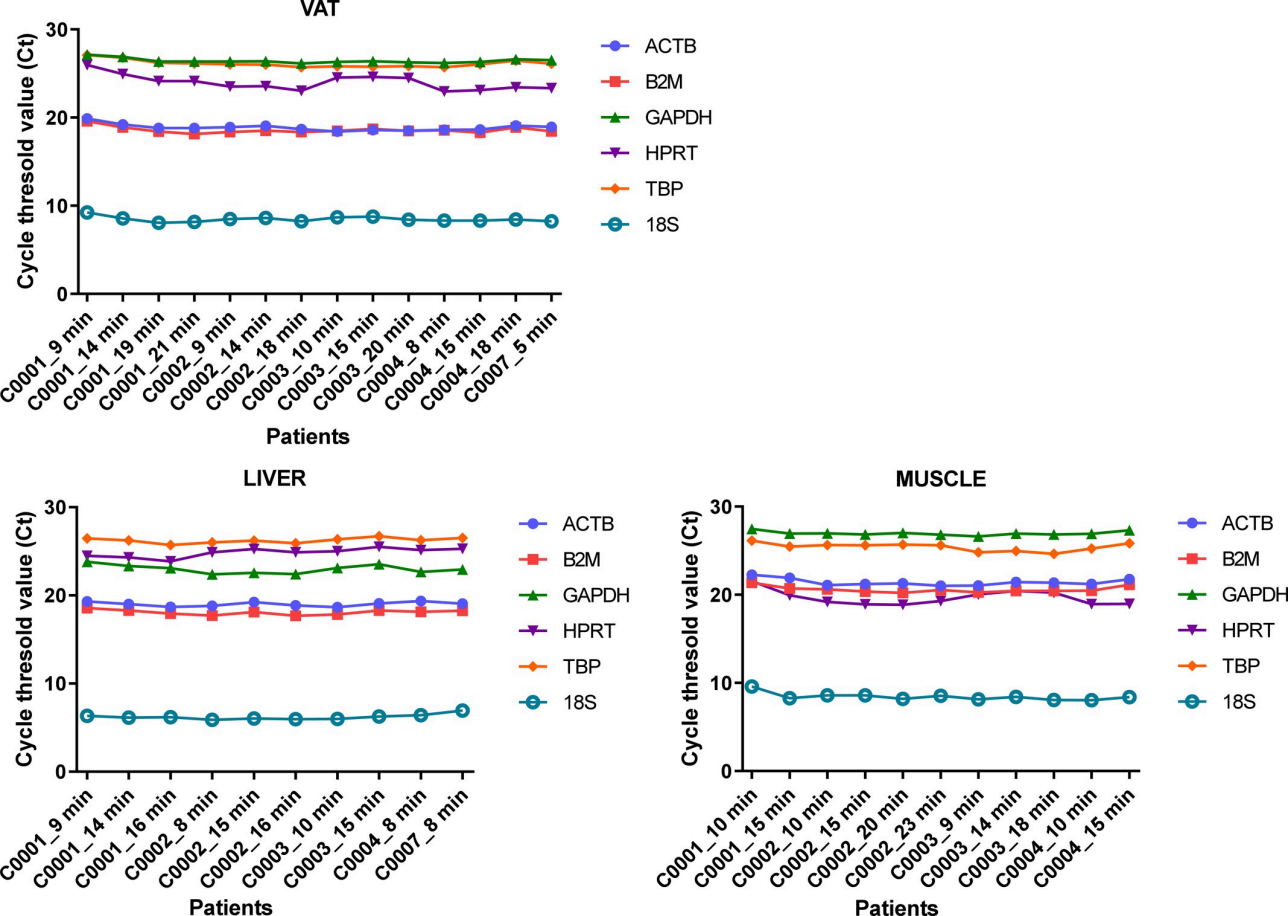
Cycle threshold (Ct) values of six housekeeping genes according to handling time for tissue preparation for the first five patients. RNA was used to perform a RT–qPCR in order to amplify six housekeeping genes (ACTB, B2M, GAPDH, HPRT, TBP, 18S). Ct values were shown for the genes according the type of tissue samples resected from the first five patients and the handling time between tissue resection and snap–freezing. VAT, visceral adipose tissue.

In order to assess the maximum handling time (ex-vivo ischemia time after biopsy) allowing a good RNA integrity, we evaluated different timing required for their aliquoting on ice (i.e. the time measured from patient surgical excision to freezing), as follows: ≤ 10, ≤ 15, ≤ 20 minutes for all samples and ≤ 25 minutes (adipose tissues only) after tissue resection. Two parameters reflecting RNA quality were evaluated during this validation and verification process: RIN and mRNA expression of classical housekeeping genes, using RT-qPCR. Concerning RIN, no significant difference in RNA samples (n = 57) could be observed for the four tissues tested and the different times of handling (Fig 3). For RT-qPCR, we selected 35 out of the 57 RNA samples (VAT, liver and muscle), that presented sufficient RNA yield and RIN over 6.7 in order to minimize the impact of RIN on mRNA expression analysis, as previously described [12]. We have also selected six housekeeping genes used as quality markers that are relevant for normalization of RT-qPCR experiments in the tissues studied. As shown in Fig 4, the six housekeeping genes were efficiently detected in all samples tested. No significant differences in intra- or inter-patient cycle threshold (Ct) among samples from the same tissue type could be observed, whatever the handling time used for sample preparation.

According to the number of samples assessed per timing, we validated maximum handling times of 15 minutes for liver and muscle and 20 minutes for adipose tissues for all future samplings. The timing determined during this validation and verification process was included in the standard procedures used for the establishment of the biobank (Table 2). When samples were not prepared within these timing, this was recorded on the Sample tracking sheet and in Tumorotek^®^.

### RNA quality over time

**Fig 5:**
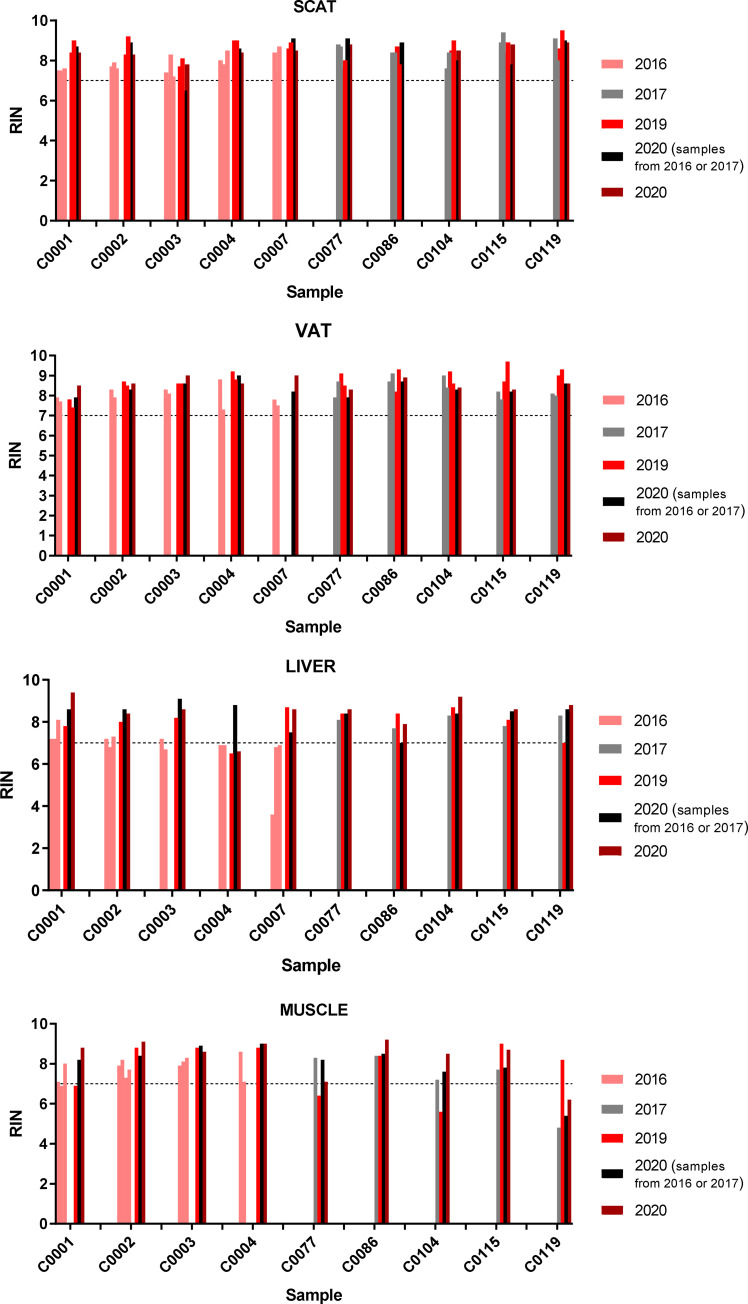
RIN according to the time of sample storage in the biobank. For each patient, RIN was shown for RNA samples 1) obtained after extraction from the four tissue types carried out in 2016 or 2017, 2) after their storage at –80°C during 3 to 4 years and re–evaluation in 2020, 3) obtained after destocking of frozen tissues from the biobank and extraction in 2019 and 2020. Results are shown for samples obtained from the first five patients included in 2016 and other patients included in 2017. SCAT, sub–cutaneous adipose tissue; VAT, visceral adipose tissue.

We also used RIN to investigate RNA quality over time on two sample types: 1) RNA samples obtained from frozen tissue stored the biobank; 2) RNA samples stored at -80°C during 3 to 4 years after extraction. Fig 5 showed RIN measured on RNA samples obtained immediately after tissue sampling in 2016 or 2017 and that of RNA samples prepared from frozen tissues after destocking from the biobank in 2019 or 2020. For each patient, RIN measured on recent RNA preparation remained in the same range as that measured on previous extraction and was even significantly improved (P<0.01 for SCAT; P<0.001 for VAT; P<0.05 for muscle; P<0.001 for liver) (Table 7). This suggests that our sample management process allowed a satisfactory conservation of frozen tissue over time. In addition, when RIN was again measured on RNA samples stored since 2016 or 2017, the values were not negatively affected by the time of RNA storage and were even slightly higher for VAT and liver (P<0.05 and P< 0.001, respectively) (Table 7). We thus propose to perform, every two years, quality controls on randomly selected samples from patients who were included every years of the project.

**Table 7:** Quality metrics of RNA isolated from frozen tissues after different time of storage in the biobank. Mean RIN ± SD were shown for RNA samples extracted from frozen tissues (SCAT, VAT, liver, and muscle) in 2016, 2017 and for RNA samples obtained from the same tissues and patient in 2019 and 2020. RNA samples extracted in 2016 and 2017 were also re–evaluated in 2020 (“redosing”). Mean DV200 ± SD were shown for RNA samples extracted in 2019 and 2020.

	Year	2016	2017	2019	2020 (redosing)	2020
	SCAT					
**RIN**	Mean ± SD	7.75 ± 0.5	8.57 ± 0.5	8.56 ± 0.5	8.56 ± 0.8	8.68 ± 0.3
**DV**_**200**_				94 ± 3		91 ± 3.8
	VAT					
**RIN**	Mean ± SD	7.89 ± 0.5	8.39 ± 0.5	8.73 ± 0.6	8.34 ± 0.35	8.5 ± 0.3
**DV**_**200**_				93 ± 2.5		91 ± 2.1
	LIVER					
**RIN**	Mean ± SD	6.83 ± 1	7.7 ± 0.3	7.98 ± 0.7	8.35 ± 0.6	8.47 ± 0.8
**DV**_**200**_				92 ± 6.15		93 ± 5.7
	MUSCLE					
**RIN**	Mean ± SD	7.76 ± 0.5	7.28 ±1.5	7.52 ±1.4	7.5 ± 1.2	7.94 ± 1.2
**DV**_**200**_				88 ± 7.8		89 ± 11.15

An additional metric, DV_200_, which represents the percentage of RNA fragments over 200 nucleotides [7], was also used to confirm RNA quality. We calculated DV_200_ using Bioanalyzer electrophoregrams for RNA samples obtained in 2019 and 2020 (Table 7). For all RNA samples, DV_200_ was between 88 and 94% and were largely over the 30% cutoff making the samples suitable for RNA sequencing. Moreover, no significant different could be observed between samples prepared in 2019 and 2020.

### Histological tissue quality over time

**Fig 6:**
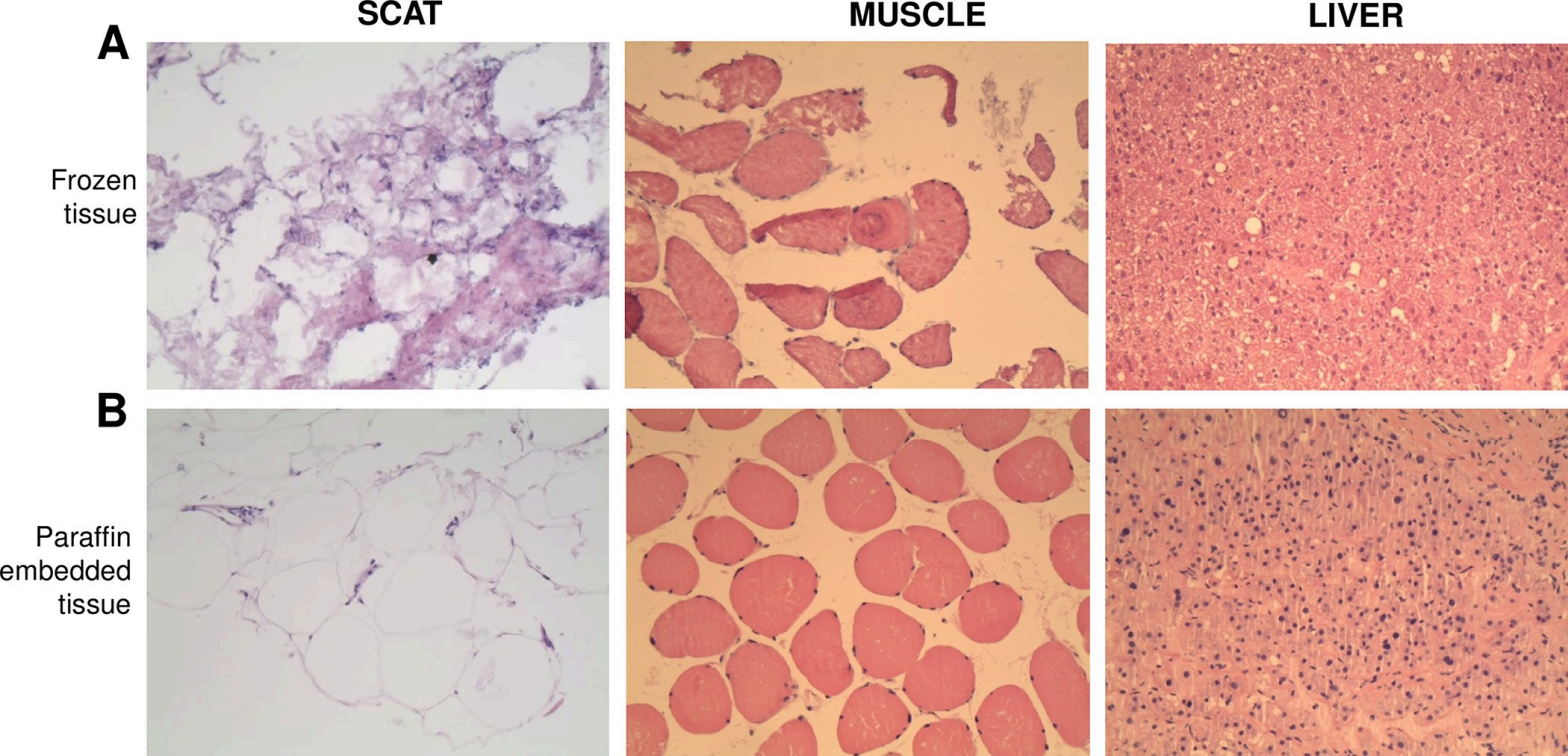
Histological integrity according to the time of sample storage in the biobank. Sections of SCAT, liver and muscle stored since 2016 were performed and stained with hematoxylin–eosine in 2020. Images were shown according to the material preparation protocol: (A) direct evaluation on frozen tissue sections (B) evaluation after the tissue was thawed, fixed and embedded in paraffin. Magnification is x200. SCAT, sub–cutaneous adipose tissue.

Histological tissue integrity was another parameter evaluated by the pathologists, to allow quality measurement over time. The pathologist takes into account the staining characteristics, tissue architecture, cell morphology and sharpness of the outlines to qualify the tissue as satisfactory or not satisfactory. Several months after storage, frozen tissue sections issued from three patients gave satisfactory histological quality except one SCAT sample, that present little analyzable material (Table 8). When tissues were thawed, fixed and embedded in paraffin, the histological quality was homogeneously satisfactory for all tissue types independently of the storage time (Table 8). Representative images of tissues (SCAT, muscle and liver from C0046 patient), according to the material preparation protocol, were shown in Fig 6.

**Table 8:** Histological integrity according to the time of sample storage in the biobank. Tissue samples from 3 patients were analyzed for their histological integrity after several months of storage in the biobank. Sections of SCAT, liver and muscle were performed directly on frozen tissues or after thawing, fixation and embedding in paraffin, and stained with hematoxylin–eosine. Histological integrity was analyzed by the pathologist under an optical microscope. S: satisfactory quality; NS: not satisfactory; ND: not determined. SCAT, sub–cutaneous adipose tissue.

Patient no.	Tissue type	Year of storage	Storage time (months)	Histological quality on frozen tissues	Histological quality after thawing and embedding in paraffin
C0046	SCAT	2016	43	**NS**	**S**
	Muscle			**S**	**S**
	Liver			**S**	**S**
C0545	SCAT	2017	29	**S**	**S**
	Muscle			**S**	**S**
	Liver			**ND**	**S**
C1500	SCAT	2019	7	**ND**	**S**
	Muscle			**ND**	**S**
	Liver			**ND**	**S**

## Discussion

Since the start of the COMET biobank, we have collected 47758 samples from 250 patients, of which 20738 were transferred to academic and industry partners. Transfers of samples take place on request of partners for their own research project. Two publications using these samples have already been published by partners [11, 13].

Here, we describe the efficiency of QMS developed for the constitution of the COMET biobank–a biocollection of human metabolic tissues collected during bariatric surgery–to guarantee the reliability and quality of biospecimens in a human tissue biobank and avoid the lack of associated data. The QA and early QC performed on samples from the first included patients enabled us to rapidly include corrections in the technical protocol to ensure sample processing reproducibility and optimize sample quality. The non-conformity rate at the time of biospecimen collection was 0,4% of total samples collected, mainly due to non-respect of sampling procedures or patient health status at that time. In that case, these samples were discarded from use. Furthermore, random periodic QCs performed on samples confirmed the maintenance of a consistent level of quality after prolonged storage at -80°C.

Concerning blood-derived samples, a unique centrifugation protocol for both serum and plasma isolation is applied to simplify and standardize the separation method. In addition, changes in the operating procedure allow isolating a higher volume of serum to be extracted: transfer at room temperature, increased centrifugation time and temperature, higher centrifugation speed and an increased number of blood tubes. Our protocol is in line with that proposed in the study of Ammerlaan et al. [14], which suggests a centrifugation at 2000g at a temperature higher than 4°C.

To obtain DNA biopsecimens, we chose to use PAXgene tube of 8.5 ml and PAXgene Blood DNA kit for DNA extraction. This allows us to isolated greater DNA yield than what reported in other biobanks using standard EDTA tubes without DNA preservative [15, 16]. This is also related to the fact that in some biobanks, whole blood samples are frozen and stored over a relatively long period prior to DNA extraction [15, 16]. The strong variability in DNA yields observed from one patient to another led us to propose the sampling of an additional PAXgene tube during the M3 or M12 visit. For all samples, the DNA is of good quality, with no signs of fragmentation even after several years of storage, in line with published data [15, 16]. As such, DNA sample management standardization is useful to obtain DNA of quality, even if genomic DNA appears less sensitive than RNA to factors affecting biospecimen quality [17]. A recent study conducted in the frame of External Quality Assurance (EAQ) program reports that DNA yields are impacted by extraction method and elution buffer, and DNA integrity by extraction method and equipment [18]. Additionally, we performed a retrospective study to analyze the impact of preanalytical factors and patient characteristics (age, sex and BMI) on the quantity of DNA extracted. We found that the storage time at 4°C before extraction impacts DNA level when it exceeds 21 days after sampling contrary to 28 days proposed by the supplier.

Regarding tissue samples,–omics profiling such as genomics, transcriptomics, proteomics and metabolomics, are widely used to generate large-scale data that can be correlated with patients’ clinical features [1]. Nonetheless, transcriptomic analyses are one of the most critical techniques since RNA can be degraded by RNases during tissue processing and nucleic acid extraction. During tissue preparation, ex-vivo warm ischemia is a major concern influencing tissue quality, and subsequent RNA integrity, even if some discrepancies arise from the literature. Indeed, some groups found that RNA remained intact up to 16 hours after resection irrespective of the storage temperature (room temperature or ice) [19], whereas others observed RNA degradation as soon as after 1 hour of cold storage [20]. The temperature at which the biopsy is maintained before snap-freezing also influences RNA integrity and the best RNA quality seemed to be obtained at 4°C [21]. In addition, RNA stability is clearly dependent on tissue type (localization, normal or tumor tissue), with some tissues being more sensitive to degradation, such as liver [20] or colon [22] (for review see [3]MA). Current biobanking practice suggests snap-freezing biospecimens within 30 minutes [23] and for more delicate tissues, such as hepatocellular carcinomas, a recent study recommends a maximum of 15 minutes, in line with the conditions we have chosen [21].

RNA extraction performed on frozen tissues resected from the first five patients, indicates that the majority of the samples had an RIN ≥ 7, a cutoff that appears sufficient for RNA microarray and expression analysis [24, 25]. This suggests that RNA isolated from these tissues can be used for downstream highly-sensitive transcriptomic profiling. For all tissue types, the A260/280 ratio reaches satisfactory values, in line with what is found in human tissues and cell lines [26], suggesting limited protein contamination. Moreover, we observe that different handling times for sample aliquoting do not impact both RNA quality and purity. This result was confirmed through analysis of mRNA expression of target genes by RT-qPCR. Indeed, we efficiently amplify widely-used housekeeping genes in all the samples tested, with no inconsistency among samples from a given patient, a given tissue or a given handling time. Thus, the results of the sample management process led us to choose maximum ex-vivo ischemia periods of 15 minutes (liver and muscle) and 20 minutes (adipose tissues), along with suitable conditions of tissue handling and transport enabling the maintenance of good RNA integrity in frozen tissues. Importantly, the sample management process allows the maintenance of tissue quality over time, as RIN from newly extracted samples remains unchanged. This is in line with Andreasson’s study showing that long-term storage at -80°C (up to three decades) does not affect RNA quality from stored tissues [27].

According to the literature, the relevance of RNA integrity as an indicator of tissue biospecimen quality and degradation can be questioned. The conventional use of the 28S/18S rRNA ratio is found to be highly variable [26, 28] and a ratio of 2.0 difficult to achieve, especially for RNA derived from human samples–which is not necessarily synonymous with poor RNA quality. Therefore, as a consequence of this lack of 28S/18S rRNA ratio reliability, RIN is now preferred and widely used for quantifying RNA quality [12, 25, 29, 30]. RT-qPCR is another method used to evaluate RNA integrity by analyzing mRNA expression of target genes. Indeed, some groups reported that ex-vivo warm ischemia can induce significant variations of gene expression patterns depending on the type of tissue and storage conditions [23], without changes in the RIN number [31]. However, concerning human biospecimen, it was previously shown that RNA quality metrics (Degradometer and RIN analysis) were predictive of gene expression analysis in RNA samples [26].

DV_200_ is another quality metric proposed by Agilent in 2014 to measure the percentage of RNA fragments above 200 nucleotides [7]. DV200 allows classifying RNA according to their size distribution and is a useful tool for degraded RNA samples having low RIN. Indeed, Matsubara et al. showed that DV200 is superior to RIN, especially for low-quality RNA, to predict efficient library production for RNA sequencing [32].

Besides RNA quality, tissue morphology can be also a relevant marker of the quality of biospecimens. The histological quality of tissue sample were satisfactory, with no traces of artefacts on frozen sections, as previously described when liver porcine biopsies are not maintained dry before freezing [20]. Thawing and fixing frozen tissue pieces rapidly alter tissue integrity [33]. Here, we confirmed that our frozen tissues can be thawed, fixed and included in paraffin for further histological analysis, as previously shown for other biospecimen [33]. For example, liver sample can be used for the diagnosis of non-alcoholic fatty liver disease (NAFLD) and non-alcoholic steato-hepatitis (NASH). Again, tissue morphology is conserved regardless of storage time, which is consistent with other data obtained on endocrine tissues [27].

## Conclusion

The quality of the samples from the first patients included in COMET has been evaluated as satisfactory. These findings therefore validate our QMS and confirm the need to implement procedures ensuring protocol compliance in order to create a high-quality human metabolic tissue collection. Linked to validated associated data, such a collection will represent an accessible resource for translational research studies in the field of T2D and metabolic disorders. Two studies that used COMET samples and data have already been published [11, 13], confirming the value of this biobank.

## Supporting information

S1 Raw image Raw image of Fig 1. (A) Electrophoresis of increasing amounts of DNA after extraction from PAXgene tube. (B) Electrophoresis of DNA after extraction and storage at -80°C. The year in which the extraction is carried out is indicated. MM: molecular markers.
(PDF)

S1 Raw data Raw data of Fig 3. RIN according to handling time for subcutaneous adipose tissue, visceral adipose tissue, muscle and liver for the first five patients.
(PDF)

S2 Raw data Raw data of Fig 4. Cycle threshold (Ct) values of six housekeeping genes according to handling time for subcutaneous adipose tissue, visceral adipose tissue, muscle and liver preparation for the first five patients.
(PDF)

S3 Raw data Raw data of Fig 5. RIN according to the time of sample storage in the biobank.
(PDF)

S1 Table List of primers used for RT-qPCR. (DOCX)
