# Supplementary material for: Addressing the quality challenge of a human biospecimen biobank through the creation of a quality management system
Source: PLoS One. 2022 Dec 30;17(12):e0278780. doi: 10.1371/journal.pone.0278780 (PMC9803146; doi:10.1371/journal.pone.0278780)

S1_raw_image: raw image of figure 1. (A) Electrophoresis of increasing amounts of DNA after extraction from PAXgene tube. (B) Electrophoresis of DNA after extraction and storage at -80°C. The year in which the extraction is carried out is indicated. MM: molecular markers.

Figure 1A

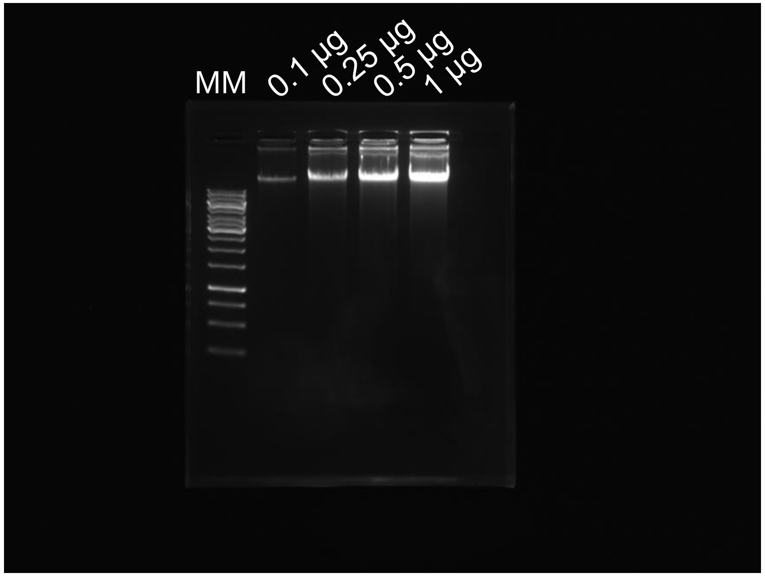

Figure 1B

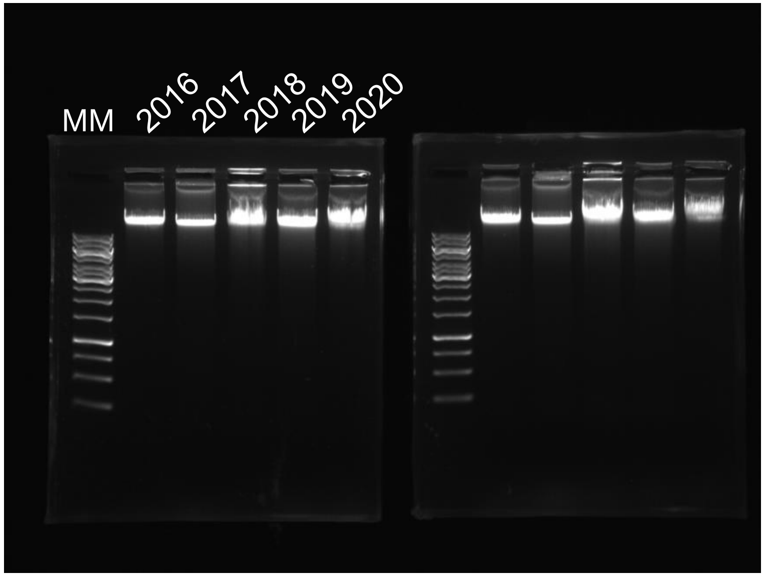

Supplement: S1 Raw image — (A) Electrophoresis of increasing amounts of DNA after extraction from PAXgene tube. (B) Electrophoresis of DNA after extraction and storage at -80°C. The year in which the extraction is carried out is indicated. MM: molecular markers. (PDF) [file pone.0278780.s001.pdf]
