# Supplementary material for: Addressing the quality challenge of a human biospecimen biobank through the creation of a quality management system
Source: PLoS One. 2022 Dec 30;17(12):e0278780. doi: 10.1371/journal.pone.0278780 (PMC9803146; doi:10.1371/journal.pone.0278780)
Supplement: S1 Raw data — RIN according to handling time for subcutaneous adipose tissue, visceral adipose tissue, muscle and liver for the first five patients. (PDF) [file pone.0278780.s002.pdf]

S2_raw_data: Raw data of figure 3. RIN according to handling time for subcutaneous adipose tissue, visceral adipose tissue, muscle and liver for the first five patients.

Subcutaneous adipose tissue

Manipulation time (minutes)	C01	C02	C03	C04	C07
1					
2					
3					
4					
5					8,4
6					
7					
8					
9			7,4		
10	7,5	7,7		7,8	7,4
11					
12					
13					
14			6,8		
15	7,5	7,9		7,8	8,7
16					
17					
18			8,3		
19					
20	7,4	7,6		8,5	
21			7,2		
22					
23					
24				8	
25	7,6				
26					
27					
29					
30					

Visceral adipose tissue

Manipulation time (minutes)

	C01	C02	C03	C04	C07
1					
2					
3					
4					
5					7,5
6					
7					
8				8,5	
9	7	8,1			
10			8,3		7,5
11					
12					
13					
14	7,1	8,3			
15			7,8	8,8	7,5
16					
17					
18		7,9		7,3	
19	7,9				
20			8,1		
21	7,7				
22					
23					
24					
25					
26					
27					
29					
30					

Muscle

Manipulation time (minutes)

	C01	C02	C03	C04	C07
1					
2					
3					
4					
5					
6					
7					
8					
9			7,9		
10	6,9	7,9		8,6	
11					
12					
13					
14			8,1		
15	8	8,2		7,1	
16					
17					
18			8,3		
19					
20	7,1	7,3			
21					
22					
23		7,7			
24					
25					
26					
27					
29					
30					

Liver

Manipulation time (minutes)

	C01	C02	C03	C04	C07
1					
2					
3					
4					
5					3,6
6					
7					
8		6,8		6,9	6,8
9	7,2				
10			7,2		
11					
12					
13					
14	7,2				
15		7,3	6,7		
16	8,1	7,2			
17					
18					
19					
20					
21					
22					
23					
24					
25					
26					
27					
29					
30					
