# Supplementary material for: Addressing the quality challenge of a human biospecimen biobank through the creation of a quality management system
Source: PLoS One. 2022 Dec 30;17(12):e0278780. doi: 10.1371/journal.pone.0278780 (PMC9803146; doi:10.1371/journal.pone.0278780)
Supplement: S2 Raw data — Cycle threshold (Ct) values of six housekeeping genes according to handling time for subcutaneous adipose tissue, visceral adipose tissue, muscle and liver preparation for the first five patients. (PDF) [file pone.0278780.s003.pdf]

S3_raw_data: raw data of figure 4. Cycle threshold (Ct) values of six housekeeping genes according to handling time for subcutaneous adipose tissue, visceral adipose tissue, muscle and liver preparation for the first five patients.

Visceral adipose tissue

	ACTB	B2M	GAPDH	HPRT	TBP	18S
C0001_9 min	19,89	19,58	27,13	25,96	27,08	9,23
C0001_14 min	19,21	18,9	26,89	24,93	26,78	8,57
C0001_19 min	18,82	18,42	26,36	24,14	26,21	8,07
C0001_21 min	18,81	18,15	26,35	24,13	26,15	8,16
C0002_9 min	18,92	18,37	26,35	23,5	26,04	8,49
C0002_14 min	19,06	18,5	26,4	23,56	26,02	8,62
C0002_18 min	18,68	18,35	26,14	23,04	25,72	8,24
C0003_10 min	18,41	18,49	26,3	24,54	25,8	8,68
C0003_15 min	18,59	18,71	26,4	24,62	25,77	8,77
C0003_20 min	18,51	18,49	26,26	24,49	25,83	8,42
C0004_8 min	18,6	18,55	26,19	22,96	25,72	8,32
C0004_15 min	18,63	18,28	26,31	23,11	26,03	8,32
C0004_18 min	19,09	18,89	26,61	23,43	26,45	8,43
C0007_5 min	18,94	18,43	26,52	23,34	26,12	8,23

Muscle

	ACTB	B2M	GAPDH	HPRT	TBP	18S
C0001_10 min	22,25	21,35	27,47	21,54	26,13	9,58
C0001_15 min	21,92	20,72	26,94	19,94	25,47	8,26
C0002_10 min	21,09	20,62	26,97	19,2	25,63	8,6
C0002_15 min	21,22	20,36	26,84	18,92	25,62	8,59
C0002_20 min	21,28	20,22	27,02	18,87	25,68	8,18
C0002_23 min	21	20,54	26,81	19,29	25,62	8,54
C0003_9 min	21,04	20,27	26,61	20,04	24,8	8,14
C0003_14 min	21,44	20,45	26,93	20,45	24,97	8,42
C0003_18 min	21,36	20,43	26,83	20,24	24,65	8,06
C0004_10 min	21,22	20,47	26,91	18,93	25,24	8,04
C0004_15 min	21,77	21,14	27,32	18,97	25,83	8,39

Liver

	ACTB	B2M	GAPDH	HPRT	TBP	18S
C0001_9 min	19,31	18,56	23,8	24,48	26,47	6,35
C0001_14 min	19	18,29	23,34	24,31	26,23	6,14
C0001_16 min	18,68	17,94	23,08	23,85	25,71	6,19
C0002_8 min	18,82	17,72	22,39	24,9	26,02	5,89
C0002_15 min	19,23	18,1	22,56	25,27	26,22	6,04
C0002_16 min	18,86	17,7	22,42	24,89	25,92	5,97
C0003_10 min	18,66	17,84	23,12	25	26,37	5,98
C0003_15 min	19,09	18,28	23,53	25,51	26,7	6,27
C0004_8 min	19,35	18,15	22,67	25,14	26,25	6,41
C0007_8 min	19,07	18,25	22,95	25,28	26,5	6,94
