# Supplementary material for: Addressing the quality challenge of a human biospecimen biobank through the creation of a quality management system
Source: PLoS One. 2022 Dec 30;17(12):e0278780. doi: 10.1371/journal.pone.0278780 (PMC9803146; doi:10.1371/journal.pone.0278780)
Supplement: S3 Raw data — RIN according to the time of sample storage in the biobank. (PDF) [file pone.0278780.s004.pdf]

S4_raw_data: raw data of figure 5. RIN according to the time of sample storage in the biobank.

Subcutaneous adipose tissue

	2016	2016	2016	2016	2017	2017	2018	2019	2019	2020	2020
C0001	7,5	7,5	7,4	7,6				8,4	9	8,7	8,4
C0002	7,7	7,9	7,6					8,3	9,2	8,9	8,3
C0003	7,4	6,8	8,3	7,2				7,7	8,1	6,5	7,8
C0004	8	7,8	7,8	8,5				9	9	8,6	8,4
C0007	8,4	7,4	8,7					8,6	8,9	9,1	8,5
C0077					8,8	8,7		7,6	8	9,1	8,8
C0086					8,4	8,4		8,7	7,8	8,9	
C0104					7,6	8,4		8,5	9	8	8,5
C0115					8,9	9,4		8,4	8,9	7,8	8,8
C0119					9,1	8		8,6	9,5	9	8,9

Visceral adipose tissue

	2016	2016	2016	2017	2017	2019	2019	2020	2020
C0001	7,1	7,9	7,7			7,8	7,4	7,9	8,5
C0002	8,1	8,3	7,9			8,7	8,5	8,3	8,6
C0003	8,3	7,8	8,1			8,6	8,6	8,6	9
C0004	8,5	8,8	7,3			9,2	8,8	9	8,6
C0007	7,5	7,5	7,5					8,2	9
C0077				7,9	8,7	9,1	8,5	7,9	8,3
C0086				8,7	9,1	8,2	9,3	8,7	8,9
C0104				9	8,4	9,2	8,6	8,3	8,4
C0115				8,2	7,8	8,7	9,7	8,2	8,3
C0119				8,1	8	9	9,3	8,6	8,6

Muscle

	2016	2016	2016	2016	2017	2019	2020	2020
C0001	7,1	6,9	8			6,9	8,2	8,8
C0002	7,9	8,2	7,3	7,7		8,8	8,4	9,1
C0003	7,9	8,1	8,3			8,8	8,9	8,6
C0004	8,6	7,1				8,8	9	9
C0077					8,3	6,4	8,2	7,1
C0086					8,4	8,4	8,5	9,2
C0104					7,2	5,6	7,6	8,5
C0115					7,7	9	7,8	8,7
C0119					4,8	8,2	5,4	6,2

Liver

	2016	2016	2016	2017	2019	2020	2020
C0001	7,2	7,2	8,1		7,8	8,6	9,4
C0002	7,2	6,8	7,3		8	8,6	8,4
C0003	7,2	6,7			8,2	9,1	8,6
C0004		6,9	6,9		6,5	8,8	6,6
C0007	3,6	6,8	6,9		8,7	7,5	8,6
C0077				8,1	8,4	8,4	8,6
C0086				7,7	8,4	7	7,9
C0104				8,3	8,7	8,4	9,2
C0115				7,8	8,1	8,5	8,6
C0119				8,3	7	8,6	8,8
