# Supplementary material for: Addressing the quality challenge of a human biospecimen biobank through the creation of a quality management system
Source: PLoS One. 2022 Dec 30;17(12):e0278780. doi: 10.1371/journal.pone.0278780 (PMC9803146; doi:10.1371/journal.pone.0278780)
Supplement: S1 Table — (DOCX) [file pone.0278780.s005.docx]

S1_table : List of primers used for RT-qPCR

| ACTB | F | GACGACATGGAGAAAATCTG |
| --- | --- | --- |
|  | R | ATGATCTGGGTCATCTTCTC |
| B2M | F | AAGGACTGGTCTTTCTATCTC |
|  | R | GATCCCACTTAACTATCTTGG |
| GAPDH | F | ACAGTTGCCATGTAGACC |
|  | R | TTTTTGGTTGAGCACAGG |
| HPRT1 | F | ATAAGCCAGACTTTGTTGG |
|  | R | ATAGGACTCCAGATGTTTCC |
| RPLP0 | F | CGGTTTCTGATTGGCTAC |
|  | R | ACGATGTCACTTCCACG |
| TBP | F | GCCAAGAGTGAAGAACAG |
|  | R | GAAGTCCAAGAACTTAGCTG |
| *F: Forward, R: Reverse* | | |
